# Haematological adaptations to high‐altitude and heat acclimation training in elite male cyclists

**DOI:** 10.1113/EP092968

**Published:** 2025-09-10

**Authors:** Claes Cubel, Magnus B. Klaris, Joakim V. Larsen, Raphaël Faiss, Lars Nybo, Carsten Lundby

**Affiliations:** ^1^ Department of Nutrition, Exercise and Sports University of Copenhagen Copenhagen Denmark; ^2^ Research and Expertise in Anti‐Doping Sciences – REDs, Institute of Sport Sciences University of Lausanne Lausanne Switzerland; ^3^ Department of Sports Science and Clinical Biomechanics, Faculty of Health Sciences University of Southern Denmark Odense Denmark

**Keywords:** altitude training, elite performance, haemoglobin mass, heat acclimation training

## Abstract

High‐altitude training is widely adopted by endurance athletes with the aim of increasing total haemoglobin mass (tHb_mass_) and thereby endurance exercise performance. However, divergent effects on tHb_mass_ and exercise performance have been reported in athletes commencing altitude camps with initial high baseline levels for tHb_mass_, questioning the efficacy of in‐season interventions in elite athletes. Therefore, haematological adaptations and exercise performance were evaluated in 12 elite cyclists completing an in‐season ‘Live High–Train High’ (LHTH) altitude camp (21 days at 3000 m) immediately after participating in the national championships. Additionally, for seven participants, we compared haematological and exercise performance effects with an off‐season heat acclimation training (HEAT) intervention (six 1‐h sessions per week for 5 weeks). The LHTH resulted in a 3.5 ± 2.0% (*P *< 0.001, *n *= 12) increase in tHb_mass_, with decay to Pre levels 10 days after returning to sea‐level. For participants followed for 9 months, the tHb_mass_ effect was comparable to that of the off‐season HEAT intervention (5.4 ± 3.9% for HEAT, LHTH vs. HEAT: *P *= 0.801, *n *= 7) and baseline levels prior to the interventions were almost identical (965 g Pre‐HEAT vs. 960 g Pre‐LHTH). Exercise performance and maximal oxygen uptake, tested immediately (2–3 days) and 10 days after LHTH, were not improved, and individual changes were not correlated to any of the haematological parameters assessed. In conclusion, the in‐season LHTH training camp effectively increased tHb_mass_ in elite cyclists; however, there was a rapid decay in tHb_mass_ upon return to sea‐level and no effect on exercise performance.

## Abstract (highlights)

- **What is the central question of this study?**
  What is the effectiveness of an in‐season ‘Live High–Train High’ (LHTH) altitude camp on haematological adaptations and sea‐level exercise performance compared to off‐season heat acclimation training (HEAT) in elite male cyclists?
- **What is the main finding and its importance?**
  The 3.5% elevation in total haemoglobin mass (tHb_mass_) following LHTH did not translate into improved exercise performance or maximal oxygen uptake at sea‐level and displayed a rapid decay after return to sea‐level. Although the boost in tHb_mass_ was similar to off‐season HEAT for participants tracked across the competitive season, the absence of an exercise performance effect is noteworthy and should be considered for athletes preparing for competitions at sea‐level.

## 1 INTRODUCTION

High‐altitude training is widely adopted by elite endurance athletes aiming at enhancing exercise performance (Bonetti & Hopkins, 2009; Garvican‐Lewis et al., 2016; Lundby et al., 2012; Millet et al., 2010; Saunders et al., 2019). Living and training at high altitude, known as ‘Live High–Train High’ (LHTH), initiates a range of physiological adaptations due to the lower barometric pressure, resulting in lower oxygen availability and hypoxia. Hypoxia activates the hypoxia‐inducible factor‐2 (HIF‐2α) cascade (Rankin et al., 2007), the master transcriptional regulator of erythropoietin (EPO) synthesis and, thereby, erythropoiesis (Montero & Lundby, 2019; Płoszczyca et al., 2018). Accordingly, EPO stimulates red blood cell synthesis, which increases total haemoglobin mass (tHb_mass_) (Garvican et al., 2012; Jelkmann, 2016; Siebenmann et al., 2015; Siebenmann, Robach et al., 2017) and the oxygen‐carrying capacity of the blood, an essential component of aerobic endurance exercise performance (Calbet et al., 2006; Schmidt & Prommer, 2010). A systematic review by Rasmussen et al. (2013) revealed that higher altitudes with consistent exposure (>20 h/day) for 3 weeks or longer periods provide a larger effect than moderate altitudes or camps with intermittent exposure (i.e. only sleeping or living at high‐altitudes for part of the day) in terms of achieving haematological adaptations. Similarly, Wilber et al. (2007) highlighted the importance of altitude in combination with total exposure time at altitude as key factors for facilitating erythropoiesis. Accordingly, studies with at least 3 weeks of LHTH (Bonne et al., 2014; Heinicke et al., 2005; Mujika et al., 2024; Rodríguez et al., 2015; Rønnestad et al., 2025) report increases in tHb_mass_. Although similar effects are reported for some ‘Live High–Train Low’ (LHTL) studies (Garvican et al., 2012; Robertson et al., 2010; Wehrlin et al., 2016, 2006), there are also studies reporting no changes in tHb_mass_ following LHTL (Siebenmann et al., 2012) or following an in‐season camp for world‐class track‐cyclists (Gore et al., 1998).

While it is clear that adequate altitude acclimatisation, or natively living at altitude, is essential for optimal exercise performance at altitude, the scientific evidence for beneficial effects on exercise performance after returning to sea‐level remains inconclusive (Bailey & Davies, 1997; Bonetti & Hopkins, 2009; Friedmann‐Bette, 2008; Lundby & Robach, 2015; Lundby et al., 2012; Millet et al., 2010). In favour of improved endurance capacity, Levine & Stray‐Gundersen (1997) reported increased ${\dot{V}}_{O_{2}\max}$ following both LHTL and LHTH training camps. However, a performance effect was observed only when the researchers made a *post hoc* division of the collegiate runners (Chapman et al., 1998). In contrast, Skattebo & Hallén (2022) determined the tHb_mass_ response in highly trained athletes completing at least five repeated high‐altitude training camps conducted at altitudes ranging from 1816 to 2320 m and could not confirm the existence of a systematic responder/non‐responder phenomenon. Instead of confirming that athletes can be classified as responders/non‐responders (i.e. systematic inter‐individual variation), it appeared that variations in tHb_mass_ gain for a given individual athlete depended on their tHb_mass_ levels ahead of the, altitude camp (i.e. intra‐athlete variation). Furthermore, Hauser et al. (2018) reported that endurance and team‐sport athletes with the highest relative tHb_mass_ (gram tHb_mass_ per kg body weight) exhibited smaller percentage increases on group level following LHTL altitude camps compared to athletes with lower relative tHb_mass_. However, some studies indicate that high initial tHb_mass_ levels prior to altitude exposure may diminish the overall effects on erythropoiesis (McLean et al., 2013; Robach & Lundby, 2012). Translation of the haematological adaptations into performance benefits is further diluted as exemplified by observations from Mujika et al. (2024) following a 4‐week LHTH training camp that effectively increased the elite swimmers’ tHb_mass_ by 5.6%, but was not associated with enhanced sea‐level swimming performance (2% lower than the swimmers’ personal records). These observations underscore the importance of evaluating and understanding the haematological and performance effects of in‐season LHTH training camps in athletes at peak physiological condition. Specifically, it remains to be investigated whether a more potent altitude stimulus, achieved by increasing altitude and thereby reducing the partial pressure of oxygen, can elicit a more pronounced tHb_mass_ increase and translate into enhanced sea‐level exercise performance in athletes already at peak physiological condition. We therefore wanted to determine the haematological and exercise performance response derived from an in‐season LHTH training camp at 3000 m in elite athletes, with thorough performance testing and tHb_mass_ monitoring before, during, immediately after and 10 days after return to sea‐level.

Prolonged heat acclimation training (HEAT) has in the last decade attracted increased attention amongst endurance athletes and been verified as an alternative intervention for increasing tHb_mass_ (Cubel et al., 2024; Lundby et al., 2023; Nybo et al., 2022; Oberholzer et al., 2019; Rønnestad et al., 2021; Rønnestad, Lid et al., 2022; Rønnestad, Urianstad et al., 2022). It has accordingly been suggested as a potential substitute for altitude training (Baranauskas et al., 2021; Nybo et al., 2022). Although the physiological stimuli are quite different, it is relevant to compare the efficiency of LHTH against HEAT from a performance perspective. In the present study, this was possible for a subgroup of participants in the LHTH study, as they also completed a 5‐week supervised HEAT intervention that involved evaluation of both the onset and decay of adaptations with tHb_mass_ and performance changes, from off‐season and throughout the majority of their competitive season.

Therefore, the present study was completed with dual aims: (1) to investigate the effects of a 21‐day in‐season LHTH training camp performed at 3000 m of altitude in elite cyclists commenced immediately after the national championships, and (2) to explore changes in tHb_mass_ and predictors of exercise performance in a subgroup of seven participants completing both an off‐season HEAT intervention and the in‐season LHTH training camp.

## 2 METHODS

### 2.1 Ethical approval

The study was approved by the ethics committee of the Capital Region of Denmark (H‐23057619). Each of the 12 participants provided their written informed consent, and the study was performed in accordance with the *Declaration of Helsinki* (World Medical Association Declaration of Helsinki, 2013). Data from the off‐season HEAT intervention involving seven participants (the HEAT group), who were also part of a larger sample (*N* = 20; *n* = 10 in HEAT and *n* = 10 in control; ethics approval no. H‐21011041), and the average responses for that study have been published in Cubel et al. (2024).However, with additional data for the subgroup of seven included in the present paper to allow for comparing changes in tHb_mass_ and exercise performance from January to August (i.e. covering both off‐ and in‐season periods).

### 2.2 Study design

**FIGURE 1:**
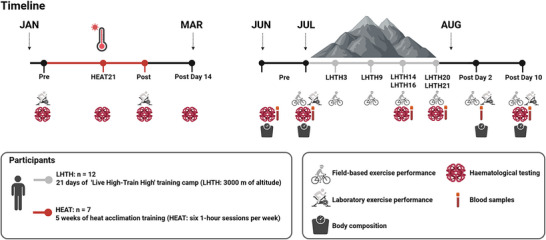
Study overview. Overall test and intervention timeline for the off‐season HEAT and the in‐season LHTH training camp intervention. Abbreviations: LHTH, ‘Live High–Train High’; HEAT, heat acclimation training; Pre, baseline; HEAT21, HEAT day 21; Post Day 14, testing 14 days after termination of HEAT; LHTH3‐21, altitude day 3 to 21; Post Day 2 and 10, testing 2–3 and 9‐11 days after returning to sea‐level. Created in https://BioRender.com.

The overall study design and testing timeline are presented in Figure 1. The altitude training intervention was designed as a 21‐day in‐season LHTH training camp at 3000 m above sea‐level in Breckenridge, CO, USA (the average barometric pressure was 540 mmHg, with a range of 535–542 mmHg, which results in a $P_{iO_{2}}$ of 103 mmHg, with a range of 102–104 mmHg), and commenced immediately after the national championships (see Figure 1). Testing of laboratory and field‐based predictors of exercise performance at sea‐level (10 m above sea‐level in Copenhagen, Denmark) was conducted 1–3 days before departure for the LHTH training camp (baseline, Pre), and again 2–3 days (Post Day 2) and 9–11 days (Post Day 10) after returning to sea‐level. Additionally, during the LHTH training camp, field‐based testing was performed on day 3 (LHTH3), 9 (LHTH9), 16 (LHTH16) and 20 (LHTH20). Haematological testing and blood sampling were performed at Pre, LHTH14, LHTH21 and Post Day 10, with additional blood sampling obtained at Post Day 2. Body composition, haematological testing and blood sampling were each conducted twice before departure at sea‐level, approximately 7 days apart, and the average of these tests was used to establish the baseline (Pre).

All training during the LHTH training camp was conducted at altitudes ranging from 2350 to 3400 m above sea‐level (the average daily temperature [training hours] was 17.5°C, with a range from 1.5°C to 22.0°C, and average humidity was 28% with a range from 15% to 51%). To ensure consistency in training and proper acclimatisation to altitude across participants with different coaches, standardised training guidelines were implemented and supervised throughout the LHTH training camp. The training progressed primarily in intensity over the 3‐week LHTH training camp. The first week was initiated with 2–3 easy days for acclimatisation, followed by a focus on low‐intensity training, supplemented with short sprints and low‐cadence torque efforts to maintain neuromuscular function. In the second week, moderate‐intensity sessions and one session of repeated short sprints were introduced (see Faiss et al., 2024). In the third week, training intensity was increased by incorporating small race simulation sessions and short high‐intensity intervals (e.g. 30/15 s; see Rønnestad et al., 2020).

The HEAT intervention consisted of 5 weeks of HEAT, comprising six 1‐h sessions per week, performed during the off‐season (January–March). Haematological testing and laboratory predictors of exercise performance testing were conducted at baseline (Pre) and after the HEAT intervention (Post). Additional haematological testing was performed after 21 days of HEAT (HEAT21) and 14 days after the termination of HEAT (Post Day 14). For a detailed description of the HEAT intervention and protocol, see Cubel et al. (2024).

Following the intervention period, participants (both for the HEAT and LHTH interventions) alternated between an oral iron supplement (13.5 mg, HemoJern, Matas Operations, Allerød, Denmark) and a multivitamin supplement (10 mg of iron, Omnimin, Biosym, Ikast, Denmark) on alternating days throughout the study to facilitate erythropoiesis.

### 2.3 Participants

Twelve male national elite cyclists (age: 24 ± 3 years; height: 184 ± 7 cm; maximal oxygen uptake [${\dot{V}}_{O_{2}\max}$]: 68.2 ± 4.5 mL min−^1^ kg^−1^; incremental peak power [IPPO]: 6.7 ± 0.5 W/kg) were included in the LHTH group, all of whom were members of the same Union Cycliste International (UCI) continental cycling team, lowlanders with no prior completion of prolonged altitude training camps. Additionally, a sub‐group of seven (age: 24 ± 2 years; height: 184 ± 8 cm; ${\dot{V}}_{O_{2}\max}$: 67.6 ± 4.0 mL min^−1^ kg^−1^; IPPO: 6.7 ± 0.5 W/kg) out of the 12 participants also completed the off‐season HEAT intervention and were followed from early January to August, as illustrated in Figure 1.

### 2.4 Experimental procedures

#### 2.4.1 Body composition and blood samples

Body composition measurements were performed using bioelectrical impedance analysis (InBody 270, InBody Co., Ltd, Seoul, South Korea). After 10 min of seated rest, a venous blood sample (∼4 mL, BD Vacutainer: K2EDTA (K2), Becton Dickinson, Franklin Lakes, NJ, USA) was drawn from an antecubital vein. The blood samples were analysed for haemoglobin concentration (Hb, g/dL) and haematocrit (%) on a haematological analyser (Sysmex XN‐1000; Sysmex, Kobe, Japan) and for bicarbonate (HCO_3_^−^, mmol/L) on a blood gas analyser (ABL800 FLEX; Radiometer, Copenhagen, Denmark).

#### 2.4.2 Testing of laboratory and field‐based predictors of exercise performance

##### Laboratory exercise performance testing

The laboratory exercise performance testing included measurements of ${\dot{V}}_{O_{2}\max}$ and IPPO. To determine ${\dot{V}}_{O_{2}\max}$ and IPPO, an incremental ramp test was performed on participants’ bikes mounted on a home trainer (Tacx Neo 2T T2875; Garmin Ltd, Olathe, KS, USA). Throughout the entire test, breath‐by‐breath measurements of gas exchange were conducted using indirect calorimetry (Vyntus CPX; Vyaire Medical, Mettawa, IL, USA) and heart rate (HR) was measured continuously with a chest strap HR monitor (HRM Dual; Garmin Ltd, Olathe, KS, USA). The ramp test begins with 3 min of on‐bike rest, followed by a 5 min warm‐up at 100 watts (W) with a cadence of 80–90 revolutions per minute (rpm). The warm‐up was followed by the ramp test, which consisted of a continuous increase in resistance at a rate of 0.5 W/s (30 W/min) until voluntary exhaustion or an inability to maintain a cadence above 70 rpm. ${\dot{V}}_{O_{2}\max}$ was defined as the highest measured 30‐s rolling average. The criteria for reaching ${\dot{V}}_{O_{2}\max}$ were determined as reaching a plateau in ${\dot{V}}_{O_{2}}$ and a respiratory exchange ratio (RER) > 1.15. IPPO was calculated by using this equation:

$$\mathrm{IPPO}=\frac{\mathrm{completion}\mathrm{time}(\mathrm{seconds})}{2}+100$$

##### Field‐based exercise performance testing

The field‐based exercise performance testing was conducted only for the LHTH group at both sea‐level (Pre and Post) and during LHTH. The field‐based testing was performed on a flat course with minimal turns to ensure optimal conditions for stable power output and HR response. All field‐based tests were conducted using the participants' private bikes, with power output measured by crank‐based power meters (Quarq; SRAM, Chicago, IL, USA). A zero‐offset calibration was performed at the start of each test day. The field‐based test protocol included a warm‐up consisting of 10 min of easy cycling. To measure peak sprint power (PSP), defined as the highest recorded power output, participants performed two 100‐m all‐out sprints. This was followed by a three‐step rate of perceived exertion test (RPE, on a scale of 1–10), which included ∼10 min at an RPE 3, ∼10 min at an RPE 5 and ∼10 min at RPE 7. After the RPE test, participants had ∼20 min of easy bicycling before completing a 20 min time trial (TT).

##### Haematological testing

To determine tHb_mass_, a carbon monoxide (CO) rebreathing procedure was performed using a semi‐automated CO‐rebreathing device (Detalo Performance; Detalo Health, Birkerød, Denmark). The methodology has been previously described in detail (Siebenmann, Keiser et al., 2017) and subsequently validated (Breenfeldt Andersen et al., 2023, 2024). To facilitate blood circulation and mixing of the administered CO, the measurement procedure was performed with participants in a supine position and with equally raised torso and legs (∼10° hip angle) throughout the entire procedure. To facilitate fingertip capillary blood sampling, the heated hand technique was applied. After 10 min of supine rest, triplets of 80 µL fingertip capillary samples were collected and immediately analysed for carboxy‐Hb (%HbCO) on a blood gas analyser (ABL800 FLEX at sea‐level and OSM3 at LHTH; Radiometer, Copenhagen, Denmark). The participants were then connected to the CO rebreathing device. Chemically pure CO (99.997%, CO N47; Air Liquide, Paris, France) was administered at a dose of 1 mL/kg body weight, and the participants breathed within a closed circuit for 6 min. During the rebreathing period, O_2_; was supplied automatically on demand. Eight minutes after the administration of the CO bolus, triplicate samples of 80 µL fingertip capillary blood were collected and immediately analysed for %HbCO. The remaining CO not absorbed by the participants during the rebreathing procedure was determined through automatic assessments of the system's volume, combined with a manual measurement of residual CO (in parts per million, ppm) in the system using a single‐gas detector (Dräger Pac 6000; Drägerwerk AG, Lübeck, Germany). Plasma volume (PV), red blood cell volume (RBCV) and total blood volume (BV) were calculated according to the method described by Siebenmann, Keiser et al. (2017), using Hb (g/dL) and haematocrit (%) values obtained from the resting blood sample. The CO‐rebreathing procedure was repeated after 10 min of breathing atmospheric air to obtain duplicate measures of tHb_mass_ during each visit. Triplicate measurements were performed in 1.7% of all sessions when the difference between duplicate tHb_mass_ values exceeded 3%. Using this protocol, the typical error for duplicate measures of tHb_mass_ was 1.1%.

##### Training data

For the LHTH group, training data were collected over 11 weeks, comprising 4 weeks at sea‐level (Pre) before altitude, 3 weeks at LHTH and 4 weeks post‐altitude at sea‐level (Post). To record training session data, a GPS bike computer (Garmin Ltd, Olathe, KS, USA and Wahoo Fitness, Atlanta, GA, USA), an HR monitor (HRM‐Dual; Garmin Ltd, Olathe, KS, USA), and crank‐based power meters (Quarq; SRAM, Chicago, IL, USA) were used. Throughout this period, the power output (in W), HR (in beats per minute), distance covered (in kilometres) and duration (in minutes) of bicycling were recorded. Before the start of each training session, a zero‐offset calibration of the power meters was performed to ensure accurate measurements. The weekly training volume was defined as the total distance covered each week. The training intensity was defined by two systems based on sea‐level exercise performance measurements: a 5‐zone (Z) intensity model based on power output during cycling (Z1: <65% IPPO, Z2: 65–85% IPPO, Z3: 85–100% IPPO, Z4: 100% IPPO–60% PSP and Z5: >60% PSP) and a 3‐Z intensity model based on based on maximal heat rate (HR_max_) values (HR‐Z1: 50–77% HR_max_, HR‐Z2: 77–87% HR_max_ and HR‐Z3: 87–100% HR_max_) corresponding to an estimation VT1 and VT2 (Seiler, 2010). All training data were uploaded to training monitoring software (TrainingPeaks; Louisville, CO, USA) and analysed using a dedicated analysis software (GoldenCheetah v. 3.6; GoldenCheetah Development Team, open‐source, available at https://www.goldencheetah.org).

### 2.5 Statistics

All data were assessed for normality using the D'Agostino and Pearson test, and residual plots were visually inspected to evaluate deviations from homoscedasticity. Sphericity was tested, and to prevent sphericity violation, a Greenhouse–Geisser correction was applied. A one‐way repeated measures ANOVA was used to assess the effect of time, and a two‐way ANOVA was used to compare the effects of the two interventions (LHTH vs. HEAT) in the subgroup. In cases of missing data, a linear mixed‐effects model was applied (see table/figure legends for sample sizes at specific time points). *Post hoc* analyses using the Bonferroni correction were performed to examine differences across time points when a significant main effect of time was identified. Statistical analyses and graph generation were performed using GraphPad Prism (version 10.4.0; GraphPad Software, Boston, MA, USA). Data are presented as means ± standard deviation (SD), and the significance level was set at *P *< 0.05.

## 3 RESULTS

### 3.1 Haematological testing and blood samples

**FIGURE 2:**
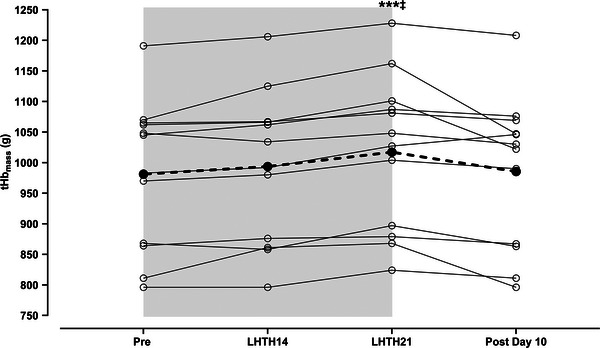
tHb_mass_ before, during LHTH and after returning to sea‐level. Values were presented as the means (filled circles and dashed line) and individual values (open circles and thin lines), with the grey area representing the LHTH training camp. A one‐way ANOVA was used for significance testing: ^***^significantly different from Pre, *P *< 0.001;^‡^significantly different from LHTH14*: P *< 0.001. Abbreviations: LHTH, ‘Live High–Train High’; tHb_mass_, total haemoglobin mass; Pre, baseline; LHTH14 and 21, altitude day 14 and 21; Post Day 10, testing 9‐11 days after returning to sea‐level.

There was an overall time effect for tHb_mass_ (*P *= 0.007; see Figure 2 for group and individual responses), with *post hoc* pairwise comparisons revealing no significant change in tHb_mass_ after 14 days (*P* = 0.292 for LHTH14 vs. Pre). However, from LHTH14 to LHTH21, there was a 24 ± 12 g (2.4 ± 1.3%, *P *< 0.001) increase in tHb_mass_, and from Pre to LHTH21, the total increase was 34 ± 18 g (3.5 ± 2.0%, *P *< 0.001). 10 days after returning to sea‐level, there was a numerical decrease of 32 ± 38 g (3.1 ± 3.5%, *P *= 0.069) from LHTH21 to Post Day 10, and the average tHb_mass_ was similar (*P *> 0.999) at Post Day 10 compared to Pre (see Table 1). At LHTH14, the PV was reduced by 460 ± 280 mL (12.2 ± 7.5%, *P *< 0.001) and accompanied by an increase in haematocrit of 3.0 ± 2.0 percentage points (6.9 ± 4.8%, *P *= 0.002; see Table 1). From LHTH14 to LHTH21, there was an increase in PV of 308 ± 258 mL (10.0 ± 8.6%, *P *= 0.010), and 10 days after returning to sea‐level (Post Day 10), a decrease of 259 ± 260 mL (7.5 ± 7.6%, *P *= 0.024) was observed compared to Pre. BV followed the same temporal patterns as PV across the tested time points. A significant reduction in BV was observed on Post Day 10 compared to LHTH21 (see Table 1), which reflects the decrease in PV. For RBCV, an increase of 86 ± 54 mL (2.8 ± 2.4%, *P *< 0.001) was observed at LHTH21 compared to LHTH14, while no changes were observed at the other time points.

**TABLE 1:** Body composition, blood samples and haematological testing.

	Pre	LHTH14	LHTH21	Post Day 2	Post Day 10	*F* (DFn, DFd), *P*‐value
Body weight (kg)	71.8 ± 6.8	—	—	70.5 ± 6.2^***^	70.7 ± 6.5^***^	*F* (1.961, 21.57) = 27.54, *P <* 0.001
Body fat (%)	11.9 ± 2.4	—	—–	12.5 ± 2.8	12.8 ± 2.4	*F* (1.723, 18.95) = 2.24, *P *= 0.139
Skeletal muscle mass (kg)	35.9 ± 3.7	—	—	34.5 ± 3.1^***^	34.7 ± 3.4^**^	*F* (1.278, 12.78) = 14.34, *P *= 0.001
HCO_3_^−^ (mmol/L)	27.0 ± 1.8	—	—	25.2 ± 1.9^*^	25.2 ± 1.8^*^	*F* (1.847, 20.31) = 9.12, *P *= 0.002
Haematocrit (%)	43.8 ± 2.7	46.8 ± 2.8^**^	45.2 ± 2.3	46.6 ± 2.2^**^	45.3 ± 2.1^†^	*F* (3.220, 35.41) = 11.40, *P *< 0.001
Hb (g/dL)	14.7 ± 1.0	16.1 ± 1.0^***^	15.5 ± 0.9^*^	15.9 ± 0.9^***^	15.4 ± 0.7^**^	*F* (2.324, 25.76) = 14.50, *P *< 0.001
tHb_mass_ (g)	983 ± 124	993 ± 124	1017 ± 126^***‡^	—	985 ± 125	*F* (1.149, 16.40) = 7.82, *P *= 0.007
PV (mL)	3757 ± 412	3297 ± 430^**^	3605 ± 370^#^	—	3498 ± 225^*^	*F* (2.483, 27.31) = 13.02, *P <* 0.001
RBCV (mL)	2919 ± 330	2890 ± 349	2976 ± 359^#^	—	2897 ± 357	*F* (1.748, 19.23) = 3.98, *P *= 0.040
BV (mL)	6676 ± 682	6187 ± 693^***^	6581 ± 662^#^	—	6395 ± 625*^§^	*F* (2.702, 29.73) = 13.49, *P <* 0.001

Values are presented as means ± SD. A one‐way ANOVA was used for significance testing: ^***^significantly different from Pre: *P *< 0.001; ^**^significantly different from Pre: *P *< 0.01; ^*^significantly different from Pre: *P *< 0.05; ^‡^significantly different from LHTH14: *P *< 0.001; ^#^significantly different from LHTH14: *P *< 0.05; ^†^significantly different from Post Day 2: *P *< 0.05; ^§^significantly different from LHTH21: *P *< 0.05. Sample size for skeletal muscle mass (kg): Pre (*n *= 12), Post Day 2 and 10 (*n* = 11). A mixed‐model analysis was used for significance testing: ***significantly different from Pre: *P *< 0.001; **significantly different from Pre: *P *< 0.01. Abbreviations: LHTH, ‘Live High–Train High’; Pre, baseline; LHTH14 and 21, altitude day 14 and 21; Post Day 2 and 10, testing 2–3 and 9‐11 days after returning to sea‐level; Hb, haemoglobin concentration; PV, plasma volume; tHb_mass_, total haemoglobin mass; BV, total blood volume; RBCV, red blood cell volume.

The Hb concentration (g/dL) displayed an increase of 1.4 ± 0.7 g/dL (9.5 ± 4.8%, *P *< 0.001) after 14 days (LHTH14), where the concentration was 0.8 ± 0.7 g/dL (5.5 ± 4.9%, *P *= 0.019) higher at LHTH21 compared to Pre. 2 days after returning to sea‐level (Post Day 2), an increase of 1.2 ± 0.7 g/dL (8.7 ± 4.7%, *P *< 0.001) was observed, and when tested 10 days after returning to sea‐level, Hb (g/dL) was increased by 0.7 ± 0.5 g/dL (4.8 ± 3.7%, *P *= 0.004) compared to Pre. A decrease in HCO_3_^−^ of 1.8 ± 1.7 mmol/L (6.7 ± 6.7%, *P *= 0.012) was observed from Pre to Post Day 2 and Post Day 10 (6.5 ± 9.9%, *P *= 0.016). A time effect was observed for body weight and skeletal muscle mass, with a decrease of 1.3 ± 0.7 kg (1.9 ± 0.7%, *P *< 0.001) in body weight, accompanied by a similar reduction in skeletal muscle mass (see Table 1). In contrast, no time effect was observed in body fat (%) (see Table 1).

### 3.2 Laboratory and field‐based predictors of exercise performance

#### 3.2.1 Laboratory predictors of exercise performance

**FIGURE 3:**
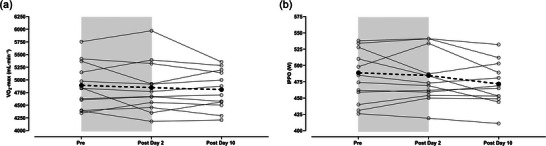
Laboratory predictors of exercise performance before and after returning to sea‐level. (a) ${\dot{V}}_{O_{2}\max}$. (b) IPPO. Values are presented as means (filled circles and dashed lines) and individual values (open circles and thin lines), with the grey area representing the LHTH training camp. A one‐way ANOVA was used for significance testing. Abbreviations: LHTH, ‘Live High–Train High’; ${\dot{V}}_{O_{2}\max}$, maximal oxygen uptake; IPPO, incremental peak power output; Pre, baseline; Post Day 2 and 10, testing 2–3 and 9‐11 days after returning to sea‐level.

**FIGURE 4:**
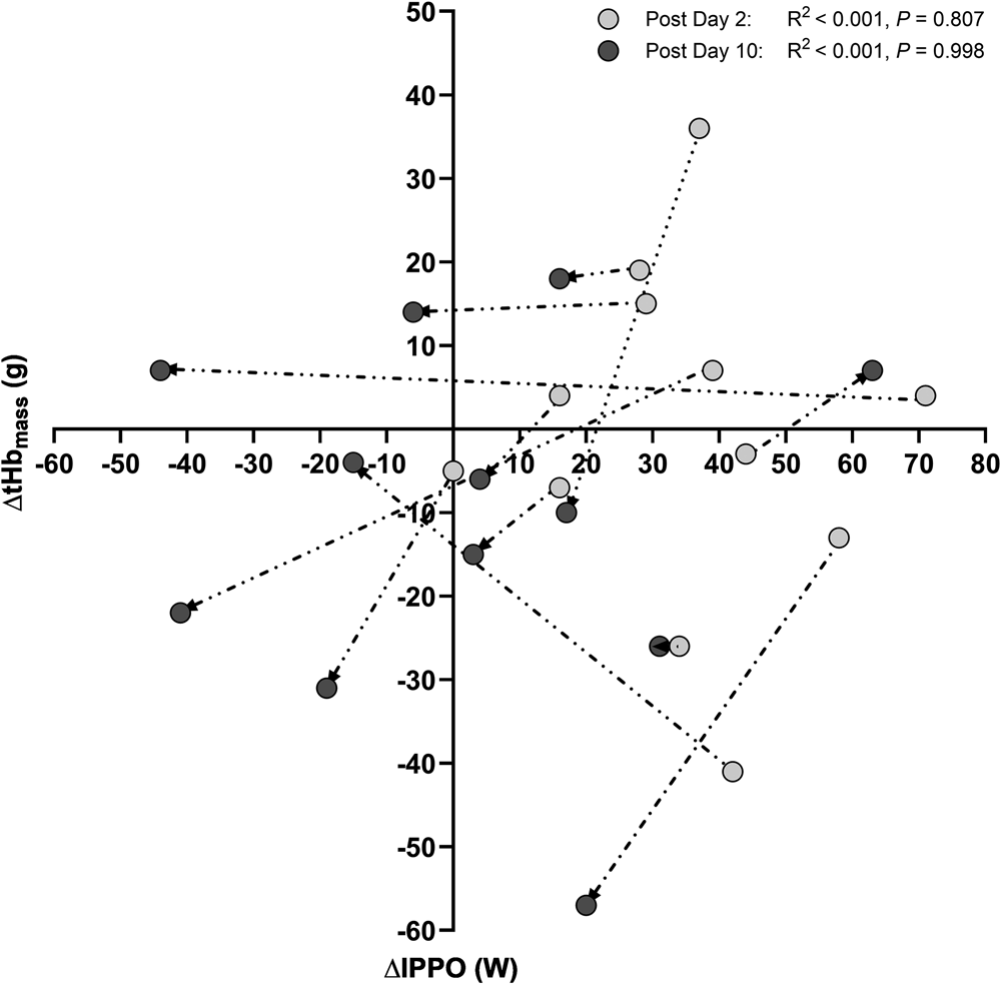
Individual delta changes in IPPO (Y‐axis) plotted against delta changes in tHb_mass_ (X‐axis) following LHTH and after returning to sea‐level. Values are presented as the delta (Δ) changes in tHb_mass_ and IPPO from Pre to Post Day 2 (light grey circles) and Post Day 10 (dark grey circles). Connecting lines (dotted) and arrows represent changes from Post Day 2 to Post Day 10. Abbreviations: ΔtHb_mass_, delta changes in total haemoglobin mass; ΔIPPO, delta changes in incremental peak power output.

For the laboratory predictors of exercise performance test, no time effect was observed for ${\dot{V}}_{O_{2}\max}$ (*P *= 0.422) or IPPO (*P *= 0.171); see Figure 3 for group and individual responses. For ${\dot{V}}_{O_{2}\max}$ and IPPO, no changes were observed at Post Day 2 (${\dot{V}}_{O_{2}\max}$: *P *= 0.902; IPPO: *P *= 0.998) and Post Day 10 (${\dot{V}}_{O_{2}\max}$: *P *= 0.351; IPPO: *P *= 0.315) compared to Pre. There was no difference between Post Day 2 and Post Day 10 (${\dot{V}}_{O_{2}\max}$: *P *= 0.920; IPPO: *P *= 0.312). The individual changes in ΔtHb_mass_ and ΔIPPO were examined Pre to Post Day 2 and from Post Day 2 to Post Day 10 after returning to sea‐level (see Figure 4). No systematic pattern was found between ΔtHb_mass_ and ΔIPPO at either Post Day 2 or Post Day 10.

#### 3.2.2 Field‐based predictors of exercise performance

All performance data from the field‐based test are presented in Table 2. No significant overall time effect was observed for PSP across the testing intervals. However, a time effect was observed when PSP was normalised to body weight (W/kg) (see Table 2). PSP (W/kg) increased at Post Day 2 (*P *= 0.023) and was significantly reduced at Post Day 10 compared to Pre (*P *= 0.008) and Post Day 2 (*P *= 0.003). For the 20‐min TT, no time effect was observed in absolute power output or when the 20‐min TT power output was normalised to body weight (W/kg) (see Table 2). In the three‐step RPE test, a time effect was observed for all variables, including HR, power output and the power‐to‐heart rate ratio (W/HR) (see Table 2). After 3 days at altitude (LHTH3), power output decreased compared to Pre and was significantly reduced following the LHTH training camp compared to Pre. Upon returning to sea‐level, power output increased when tested at Post Day 2 compared to LHTH3 but remained below Pre levels. When tested 10 days after returning to sea‐level (Post Day 10), power output had further increased but did not fully return to Pre levels. HR and W/HR exhibited a similar temporal pattern across the RPE steps (see Table 2). However, only a significant reduction in HR was observed at LHTH20 for RPE 3 (*P *= 0.046) and on Post Day 10 for multiple RPE steps (RPE 3: *P *= 0.005, RPE 5: *P *= 0.035, and RPE 7: *P *= 0.036). No changes were observed at the other time points (see Table 2).

**TABLE 2:** Field‐based predictors of exercise performance before, during LHTH and after returning to sea‐level.

	Pre	LHTH3	LHTH9	LHTH16	LHTH20	Post Day 2	Post Day 10	*F* (DFn, DFd), *P*‐value
PSP (W)	1253 ± 189	1249 ± 200	1261 ± 197	1308 ± 205	1228 ± 221	1275 ± 214	1150 ± 200	*F* (0.062, 0.583) = 4.84, *P *= 0.108
PSP (W/kg)	17.4 ± 1.9	—	—	—	—	17.9 ± 2.0^*^	16.3 ± 2.2^**†^	*F* (0.896, 8.517) = 18.67, *P *= 0.026
20 min TT (W)	386 ± 40	—	—	—	—	364 ± 19	375 ± 32	*F* (1.906, 17.15) = 2.13, *P *= 0.150
20 min TT (W/kg)	5.3 ± 0.4	—	—	—	—	5.2 ± 0.4	5.4 ± 0.2	*F* (1.988, 17.89) = 1.01, *P *= 0.383
RPE 3 (W)	278 ± 17	235 ± 28^***^	237 ± 24^***^	231 ± 33^**^	235 ± 27^***^	258 ± 30	258 ± 21^*^	*F* (3.028, 30.79) = 13.57, *P *< 0.0001
RPE 5 (W)	325 ± 27	267 ± 28^***^	268 ± 27^***^	264 ± 30^***^	267 ± 26^**^	298 ± 35^*^	303 ± 17^*^	*F* (2.761, 27.61) = 22.08, *P *< 0.0001
RPE 7 (W)	370 ± 34	286 ± 31^***^	293 ± 23^***^	299 ± 29^***^	300 ± 24^***^	345 ± 31	353 ± 22	*F* (2.761, 27.61) = 22.08, *P *< 0.0001
RPE 3 (HR)	154 ± 12	154 ± 9	154 ± 8	148 ± 10	145 ± 9^*^	147 ± 9	140 ± 8^**^	*F* (2.588, 24.31) = 45.89, *P *< 0.001
RPE 5 (HR)	164 ± 12	166 ± 7	164 ± 8	159 ± 9	156 ± 10	159 ± 8	153 ± 9^*^	*F* (3.783, 38.46) = 6.39, *P *< 0.001
RPE 7 (HR)	171 ± 7	174 ± 6	173 ± 7	169 ± 7	168 ± 11	171 ± 8	166 ± 6^*^	*F* (2.604, 24.74) = 3.14, *P *= 0.049
RPE 3 (W/HR)	1.8 ± 0.2	1.5 ± 0.2^***^	1.5 ± 0.2^****^	1.6 ± 0.2^**^	1.6 ± 0.2^*^	1.8 ± 0.2	1.9 ± 0.2	*F* (3.011, 33.11) = 22.28, *P *< 0.0001
RPE 5 (W/HR)	2.0 ± 0.2	1.6 ± 0.2^***^	1.6 ± 0.2^****^	1.7 ± 0.2^***^	1.7 ± 0.2^*^	1.9 ± 0.2^*^	2.0 ± 0.2	*F* (1.986, 19.86) = 33.47, *P *< 0.0001
RPE 7 (W/HR)	2.2 ± 0.2	1.6 ± 0.2^***^	1.7 ± 0.2^***^	1.8 ± 0.2^***^	1.8 ± 0.2^***^	2.0 ± 0.2	2.1 ± 0.2	*F* (1,945, 18.48) = 41.29, *P *< 0.0001

Values are presented as means ± SD. A mixed‐model analysis was used for significance testing: ^***^significantly different from Pre: *P *< 0.001; ^**^significantly different from Pre: *P *< 0.01; ^*^significantly different from Pre: *P *< 0.05; ^†^significantly different from Post Day 2: *P *< 0.05. Sample size included in the statistical analysis for PSP (W): Pre (*n* = 10), LHTH3–9 (*n* = 11), LHTH16 (*n* = 9), LHTH20 (*n* = 10), Post Day 2 (*n* = 11) and Post Day 10 (*n* = 10); PSP (W/kg): Pre (*n* = 12), Post Day 2 (*n* = 11) and Post Day 10 (*n* = 10); 20 min TT (W) and 20 min TT (W/kg): Pre (*n* = 11), Post Day 2 (*n* = 10) and Post Day 10 (*n* = 11); RPE 3 (W), (HR) and (W/HR): Pre (*n* = 11), LHTH3 (*n* = 10), LHTH9–20 (*n* = 12), Post Day 2 (n = 12) and Post Day 10 (*n* = 10); RPE 5 (W), (HR) and (W/HR): Pre (*n* = 11), LHTH3 (*n* = 10), LHTH9–16 (*n* = 12), LHTH20 (*n* = 11), Post Day 2 (*n* = 12) and Post Day 10 (*n* = 10); RPE 7 (W), (HR) and (W/HR): LHTH3 (*n* = 9), LHTH9 (*n* = 10), LHTH16 (*n* = 12), LHTH20 (*n*n = 11), Post Day 2 (*n* = 12) and Post Day 10 (*n* = 10). Abbreviations: LHTH, ‘Live High–Train High’; Pre, baseline; LHTH3–20, altitude day 3–20; Post Day 2 and 10, testing 2–3 and 9‐11 days after returning to sea‐level; PSP, peak sprint power; 20 min TT, 20 min time trial; RPE, rating of perceived exertion; HR, heart rate in beat/min; W/HR, watts per heart rate.

#### 3.2.3 Training

**FIGURE 5:**
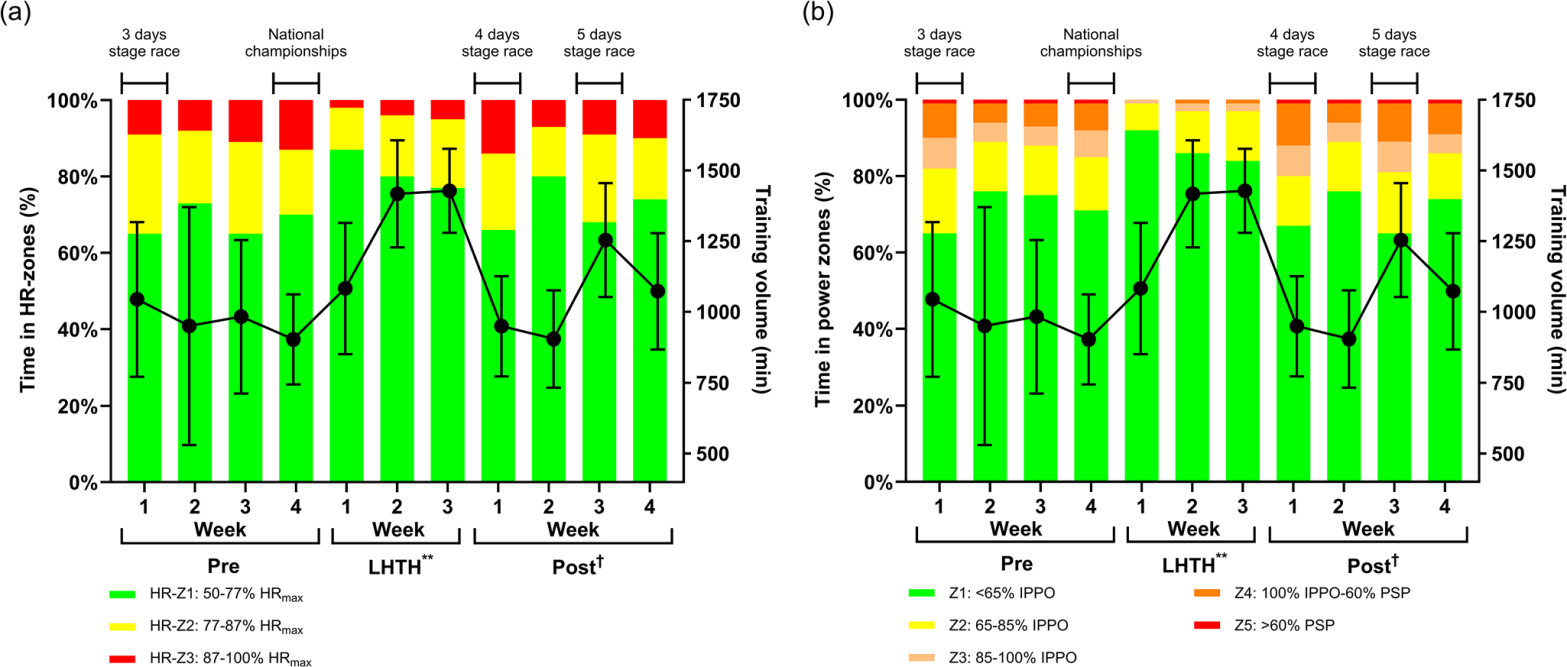
Training data overview for HR and power distribution relative to training volume before, during LHTH and after returning to sea‐level. (a) Relative distribution of time spent in heart rate zones (HR‐zones) relative to training volume (filled circles and line). (b) Relative distribution of time spent in power zones (bars) relative to training volume. Values are expressed as HR‐zone and power zone (bars) distributions, while training volume is expressed as the mean ± SD (lines and dots). A mixed‐models analysis was used for significance testing: ^**^training volume significantly different from Pre: *P *< 0.01; ^†^training volume significantly different from LHTH: *P *< 0.05. The sample size included in the statistical analysis of HR‐zones: Pre (*n* = 10), LHTH (*n* = 12), Post (*n* = 11), Week 1–3 (*n* = 12). Sample size included in the statistical analysis of power zones: Pre (*n* = 9), LHTH (*n* = 12), Post (*n* = 11), Week 1–3 (*n* = 12). Abbreviations: LHTH, ’Live High–Train High’; Pre, time at sea‐level before LHTH; Post, time following the return to sea‐level; HR‐Z1–3, heat rate zones 1–3; HR_max_, maximal heart rate; PSP, peak sprint power; IPPO, incremental peak power output.

The distribution of time spent in training zones during the intervention period is illustrated in Figure 5, which also includes the number of races completed. Figure 5a represents the time spent in HR‐zones relative to training volume (filled circles and line), while Figure 5b represents the time spent in power zones relative to training volume (filled circles and line). A time effect (*P *= 0.002) was observed f, with training volume increasing during LHTH compared to Pre (339 ± 93 min, *P *= 0.002) and decreasing during Post compared to LHTH (264 ± 91 min, *P *= 0.017). There was no difference in training volume between Pre and Post (*P *= 0.700). During LHTH, the participants, on average, spent relatively less time in power Z1 and Z3 and less time above 87% of maximum HR (HR‐Z3) compared to Pre.

### 3.3 Changes in tHb_mass_ and laboratory predictors of exercise performance with HEAT and LHTH interventions

**FIGURE 6:**
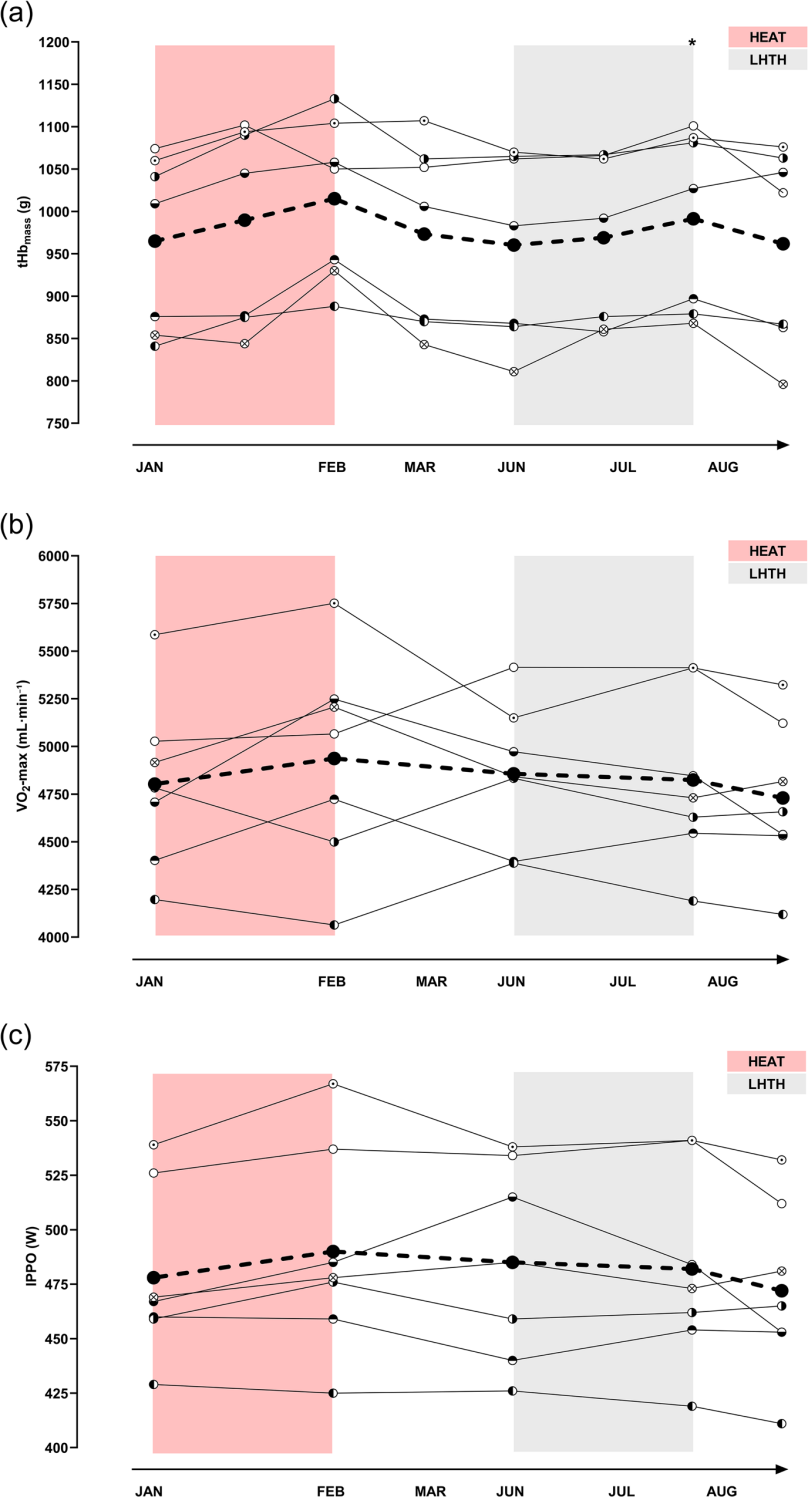
Changes in tHb_mass_ and laboratory predictors of exercise performance with HEAT and LHTH interventions. (a) tHb_mass_. (b) ${\dot{V}}_{O_{2}\max}$. (c) IPPO. Values are expressed as the mean (filled circles and dashed lines) and individual values (different circle patterns and thin lines for *n* = 7), with the red area representing the off‐season HEAT, and the grey area representing the LHTH training camp. A one‐way ANOVA was used to evaluate overall time effects, whereas a two‐way ANOVA was used to compare the effects of the two interventions (LHTH vs. HEAT): ^*^significantly different from Pre‐LHTH: *P *< 0.05. Abbreviations: HEAT, heat acclimation training; LHTH, ‘Live High–Train High’; tHb_mass_, total haemoglobin mass; ${\dot{V}}_{O_{2}\max}$, maximal oxygen uptaken; IPPO, incremental peak power output.

The changes over 9 months are presented in Figure 6 for tHb_mass_ and laboratory predictors of exercise performance. There was an overall time effect (*P *= 0.005) for tHb_mass_, and the response following the off‐season HEAT intervention and the in‐season LHTH training camp was similar from Pre to Post (HEAT: numerical change of 50 ± 37 g, 5.4 ± 3.9%, *P *= 0.105; LHTH: 31 ± 16 g, 3.3 ± 2.0%, *P *= 0.023; LHTH vs. HEAT: *P *= 0.801). Evaluation of tHb_mass_ performed 14 days after termination of HEAT and 10 days after returning to sea‐level revealed a similar rapid decay in tHb_mass_ (HEAT: 973 ± 107 g, numerical decrease of 42 ± 37 g, 4.2 ± 3.8%, *P *= 0.222; LHTH: 962 ± 117 g, numerical decrease of 29 ± 35 g, 3.1 ± 3.7%, *P *= 0.660) bringing it back to Pre levels (HEAT: 965 ± 103 g vs. LHTH: 960 ± 103 g, *P *> 0.999).

For laboratory predictors of exercise performance testing, there was no overall time effect for ${\dot{V}}_{O_{2}\max}$ (*P *= 0.345), and no changes in ${\dot{V}}_{O_{2}\max}$ from Pre to Post (HEAT: numerical change of 134 ± 262 mL min^−1^, 2.8 ± 5.6%, *P *= 0.774; LHTH: Post Day 2: numerical decrease of 33 ± 165 mL min^−1^, 0.8 ± 3.4%, *P *= 0.994; and Post Day 10: numerical decrease of 127 ± 221 mL min^−1^, 2.6 ± 4.3%, *P *= 0.655). The Pre‐${\dot{V}}_{O_{2}\max}$ was similar before the initiation of the two interventions, with no difference between (HEAT: 4803 ± 450 mL min^−1^ vs. LHTH: 4857 ± 357 mL min^−1^, *P *= 0.991). There was no overall time effect (*P *= 0.138) for IPPO, and no changes in IPPO from Pre to Post (HEAT: 489 ± 44 W, numerical change of 11 ± 12 W, 2.2 ± 2.2%, *P *= 0.178; LHTH: Post Day 2: 482 ± 42 W, numerical decrease of 3 ± 15 W, 0.5 ± 2.8%, *P *= 0.987; and Post Day 10: 472 ± 37 W, numerical decrease of 13 ± 23 W, 2.4 ± 4.6%, *P *= 0.706) The Pre‐IPPO was similar before the initiation of the two interventions, with no difference between (HEAT: 478 ± 36 W vs. LHTH: 485 ± 45 W, *P *= 0.938).

## 4 DISCUSSION

The present study demonstrates that a 21‐day in‐season LHTH training camp effectively elevated tHb_mass_ in elite male cyclists, commenced immediately after a highly prioritised competition. However, the increase in tHb_mass_ did not transfer into improved exercise capacity or field‐based predictors of exercise performance test completed immediately after (2–3 days), or 10 days upon return to sea‐level. No individual patterns were observed regarding the increase in tHb_mass_ and exercise performance after returning to sea‐level. Notably, a rapid decay in tHb_mass_ was observed 10 days after returning to sea‐level, bringing tHb_mass_ back to Pre‐LHTH levels. In a subgroup of seven participants, both the off‐season HEAT intervention and the in‐season LHTH training camp showed similar responses in tHb_mass_. However, no systematic response in laboratory predictors of exercise performance was observed.

The observed increase in tHb_mass_ following the LHTH training camp supports our initial hypothesis that providing a sufficient hypoxic stimulus through extended exposure by living and training at altitudes from 2350 to 3400 m can induce an increase in tHb_mass_ in elite athletes in‐season following a prioritised competition. These findings align with previous research demonstrating the dose–response relationship between hypoxic exposure and erythropoietic stimulation (Garvican‐Lewis et al., 2016; Gore et al., 2013; Koivisto‐Mørk et al., 2021; Rasmussen et al., 2013; Skattebo & Hallén, 2022). The observed 3.5% increase in tHb_mass_ falls within the range observed in other LHTH training camp studies (conducted at approximately 2000 m of altitude) involving endurance‐trained athletes (Garvican et al., 2012; Gore et al., 2013). Additionally, the present study protocol incorporated the CO‐rebreathing procedure, which has high precision for detecting small changes in tHb_mass_ (Breenfeldt Andersen et al., 2024; Siebenmann, Keiser et al., 2017). However, despite the increase in tHb_mass_, no transfer effect on exercise performance was observed in either laboratory or field‐based tests immediately (2–3 days) or 10 days after returning to sea‐level. Thus, within the timeframe studied, the increase in ΔtHb_mass_ was not directly associated with changes in ΔIPPO when tested 2 and 10 days after returning to sea‐level (see Figure 4). These results suggest that changes in ΔtHb_mass_ following the LHTH training camp did not correlate with changes in IPPO, regardless of the time elapsed since returning to sea‐level. It is, therefore, essential to consider the optimal timing for post‐altitude exercise performance testing to accurately assess whether there are carryover effects of altitude training camps on sea‐level exercise performance (Chapman et al., 2014; Millet et al., 2010). Several factors can influence physiological performance upon returning to sea‐level, including the quality of training before, during and after the camp, as well as post‐camp fatigue, such as jet lag and travel fatigue between two continents (Janse van Rensburg et al., 2021). As illustrated in Figure 5, training volume increased during the LHTH training camp, while participants spent less time in the higher power zones and HR‐Z3. The reduction in absolute power output observed during training and field‐based testing at the LHTH training camp may explain why no changes in sea‐level exercise performance were observed. Geiser et al. (2001) found that while training intensity did not significantly impact ${\dot{V}}_{O_{2}\max}$ improvements in normoxic or hypoxic conditions, it positively influenced maximal power output. Interestingly, cardiovascular stress and muscle oxidative capacity were not considerably affected by training intensity, suggesting these factors may be less responsive to intensity than other performance metrics. Similarly, Wehrlin and Hallén (2006) found a linear decline in ${\dot{V}}_{O_{2}\max}$ and endurance exercise performance with increasing altitude, primarily due to reduced oxygen availability. In complementary analysis, Lundby et al. (2007) found no changes in muscle mechanical efficiency following acclimatisation to moderate to high altitudes, indicating that muscle efficiency may be unaffected by altitude exposure. These findings suggest that factors other than muscle efficiency may explain the lack of performance improvements upon returning to sea‐level. While altitude training may impact specific performance metrics, power output and muscle efficiency may not change as much. In the present study, the return travel to Europe, with an 8‐h time difference, may have skewed the performance data towards the negative. Although measures of exercise performance and power output provide valuable insights into physiological adaptations relevant to elite cycling performance, they may also be affected by residual fatigue. Including systematic monitoring of subjective fatigue, sleep duration, or quality in future studies could improve the ability to distinguish the effects of residual fatigue when assessing adaptations to altitude or HEAT interventions.

To the authors’ knowledge, the present study is the first to compare the effects of HEAT and LHTH interventions within the same individuals. In the subgroup analysis, only the LHTH demonstrated a significant increase in tHb_mass_; nevertheless, the two interventions are comparable in their average response regarding tHb_mass_. However, for the HEAT intervention, when the seven participants were part of a larger sample size (*n* = 10), the tHb_mass_ response to HEAT was significant (see Cubel et al., 2024). After exposure to HEAT or LHTH was terminated, a reduction in tHb_mass_ was observed after 10–14 days. The rapid decay in tHb_mass_ is in line with previous research, which shows that removing exposure to either HEAT or hypoxia results in a decrease in tHb_mass_ within 7 days to 3.5 weeks (Cubel et al., 2024; Heinicke et al., 2005; Klein et al., 2021; Rønnestad et al., 2025; Siebenmann et al., 2015). It can be hypothesised from a theoretical point of view that using a maintenance intervention to preserve the increase in tHb_mass_ can potentially prolong the ‘time window’ during which the increase in tHb_mass_ may translate into an enhancement in sea‐level exercise performance. Recent research has demonstrated that after an LHTH training camp or a HEAT period, the rapid decrease in tHb_mass_ can be mitigated by incorporating three weekly HEAT sessions over a ∼3‐week period as a maintenance intervention (Rønnestad et al., 2025; Rønnestad, Urianstad et al., 2022). The findings of the two studies suggest that this approach not only preserves the increased tHb_mass_ but may also lead to further increases in tHb_mass_ (Rønnestad et al., 2025; Rønnestad, Urianstad et al., 2022). In addition to HEAT, other interventions, such as intermittent hypoxic exposure and sleeping in normobaric hypoxia, have been proposed as potential strategies to maintain haematological adaptations following altitude training (Millet et al., 2010). However, their efficacy and feasibility in elite sports settings remain uncertain and further research is required.

The ability to follow elite athletes over prolonged periods and prescribe interventions in‐season between the present group of riders and two main (prioritised) competitions (national championships and Tour of Denmark stage race) involved some compromises in the study design. Ideally, a control group should have been included in the LHTH intervention. The inclusion would have enabled the comparison of changes in tHb_mass_ and exercise performance among participants who undertook the same training volume and intensity distribution in HR and power output at sea‐level. Nevertheless, the observed increase in tHb_mass_ aligns with previous LHTH (Bonne et al., 2014) and LHTL (Hauser et al., 2016; Wehrlin et al., 2006) research, including a control group at sea‐level. It would have been ideal to incorporate equal sample sizes with identical participants to compare the effects of the HEAT and LHTH interventions. However, working with elite‐level athletes can be challenging due to logistical constraints, training camps and competition schedules. The HEAT intervention did not exert significant changes in IPPO, whereas such changes were observed when the participant was part of a larger sample size (*n* = 10) (see Cubel et al., 2024). Additionally, Nybo et al. (2022) reported an average increase of approximately 3% in peak power output after HEAT interventions. This study included only male elite cyclists, which ensured a homogeneous sample; however, this limits the generalisability of the findings to female athletes. The haematological and exercise performance responses to both HEAT and LHTH in elite female cyclists remain under‐researched, and future studies are needed to explore potential sex‐specific adaptations. Additionally, future research could investigate the effects of various maintenance interventions on tHb_mass_ decay and retention, as well as their long‐term influence on exercise performance following altitude or HEAT exposure. Moreover, the optimal performance ’window’ upon returning to sea‐level remains unclear; clarifying the timing of post‐altitude performance testing may be crucial for accurately capturing potential carryover effects.

In conclusion, this study demonstrates that an in‐season LHTH training camp can increase tHb_mass_ in elite cyclists already at peak physiological condition. However, the elevated tHb_mass_ did not translate into improved exercise performance at sea‐level, either immediately or several days after returning to sea‐level. In the subgroup of seven participants, both the off‐season HEAT intervention and the in‐season LHTH training camp induced similar responses in tHb_mass_, while no systematic changes in exercise performance were observed.

## AUTHOR CONTRIBUTIONS

Claes Cubel, Magnus B. Klaris, Raphaël Faiss, Lars Nybo and Carsten Lundby were involved in the experimental conception and design. Claes Cubel, Magnus B. Klaris, Joakim V. Larsen, Raphaël Faiss, Lars Nybo and Carsten Lundby conducted the study. Claes Cubel, Magnus B. Klaris, Joakim V. Larsen and Raphaël Faiss analysed and interpreted the data. Claes Cubel, Lars Nybo and Carsten Lundby drafted the manuscript and critically revised the manuscript. All authors have read and approved the final version of this manuscript and agree to be accountable for all aspects of the work in ensuring that questions related to the accuracy or integrity of any part of the work are appropriately investigated and resolved. All persons designated as authors qualify for authorship, and all those who qualify for authorship are listed.

## CONFLICT OF INTEREST

Carsten Lundby is the Chief Executive Officer (CEO) at Detalo Health, Denmark, the company responsible for manufacturing the automated CO‐rebreathing system Detalo Performance™. The other authors declare no conflicts of interest.

## Data Availability

The data supporting this study's findings are available from the corresponding author, Claes Cubel, upon request.
